# CT acquisition protocols for lung cancer screening—current landscape and the urgent need for consistency

**DOI:** 10.1186/s13244-025-01949-z

**Published:** 2025-03-26

**Authors:** Mathis Franz Georg Konrad, Emily Nischwitz, Aad van der Lugt, Gudrun Zahlmann, Viktoria Palm, Joanna Chorostowska-Wynimko, Helmut Prosch, James L. Mulshine, Hans-Ulrich Kauczor

**Affiliations:** 1 Department of Diagnostic and Interventional Radiology, Heidelberg University Hospital, Heidelberg, Germany; 2 Translational Lung Research Center Heidelberg (TLRC), German Center for Lung Research (DZL), Heidelberg, Germany; 3 Department of Radiology & Nuclear Medicine, Erasmus MC – University Medical Center Rotterdam, Rotterdam, Netherlands; 4 Independent Consultant for Quantitative Imaging Biomarkers Alliance (QIBA), Radiological Society of North America (RSNA), Oak Brook, Illinois USA; 5 Department of Diagnostic and Interventional Radiology with Nuclear Medicine, Thoraxklinik, Heidelberg University Hospital, Heidelberg, Germany; 6 Department of Genetics and Clinical Immunology, National Institute of Tuberculosis and Lung Diseases, Warsaw, Poland; 7 Department of Biomedical Imaging and Image-guided Therapy, Medical University of Vienna, Vienna, Austria; 8 Rush University, Chicago, Illinois USA

## Abstract

- Standardizing CT acquisition protocols reduces radiation exposure in lung cancer screening.
- Cross-continent collaboration will enhance understanding of diverse clinical practices.
- Survey results will inform future advancements in radiology sustainability efforts.

## To the Editor-in-Chief,

Sustainability in radiology was the focus of the European Congress of Radiology (ECR) in 2025. A survey across Europe and North America regarding CT acquisition protocols in lung cancer screening (LCS) could play a significant role in supporting sustainability efforts.

## Here’s why

In the European Union, a conservative estimate suggests that approximately 20% of adults aged 50–74 may be eligible for LCS as current or former smokers. Even with a low participation rate of 5%, this could result in over 1.4 million procedures annually [1] (halve that for biennial screening). Given this scale, even minor improvements in the application of CT acquisition protocols, which are often already considered ‘low-dose,’ could lead to substantial reductions in radiation exposure and energy consumption.

A widespread survey exploring institutional and technical factors of CT acquisition protocols for CT LCS has not yet been conducted, and this information gap needs to be filled. We are planning on implementing this survey to understand the current state of play and the survey itself may be valuable for sustainability in several ways.

The recently published results from the randomized NELSON LCS trial have demonstrated a significant reduction in lung cancer mortality. This trial utilized quantitative CT imaging, enabling high sensitivity and specificity for early lung cancer detection [2]. By employing non-invasive CT-generated measurements of nodule growth, the trial reduced the need for invasive diagnostic procedures to assess malignancy, thereby improving the safety of the screening process for participants.

NELSON was the first cancer screening trial to successfully implement safe, efficient, and effective clinical management using advanced computational image analysis. As computational analysis of CT images is expected to be a core aspect of CT screening moving forward, standardization of image acquisition and analysis will be critical quality measures as the screening process matures.

## Social sustainability

With the screening participant, and thereby potential future patient, in mind, the survey can contribute to social sustainability aspects. Understanding variations in CT protocols across institutions will help identify best practices for minimizing radiation exposure while enhancing patient safety and well-being. The survey results can serve as a basis for standardizing CT protocols across facilities, thus ensuring more consistent and equitable care for all patients [3].

## Environmental sustainability

Optimizing CT acquisition protocols can lead to reduced radiation exposure, which has possible implications for environmental sustainability. Optimized protocols often require less radiation, potentially reducing the energy needed to operate CT devices. This would lead to decreased electricity usage and associated carbon emissions. The equipment lifespan may be extended by using lower radiation doses, as CT devices may experience less wear and tear, thereby potentially extending their operational life and reducing the need for frequent replacements.

## Economic sustainability

Optimized CT acquisition protocols can contribute to economic sustainability in healthcare. These protocols can make LCS more cost-effective by reducing the resources required per scan while maintaining diagnostic accuracy [4]. In addition to the aforementioned potential device-related savings, minimizing unnecessary radiation exposure may lead to fewer radiation-induced health issues. In the context of LCS, which is limited to older individuals and exclusively uses low-dose CT imaging, the potential health risks from radiation exposure are minimal. These risks are largely considered theoretical, though continued vigilance is warranted.

The use of CT volumetry with reduced reliance on invasive diagnostic procedures could lower long-term healthcare costs. However, it is crucial to define the optimal balance between maximal dose reduction and preserving sufficient image resolution. This balance forms the foundation of thoracic CT imaging, ensuring reliable monitoring of nodule changes [5]. Cost-effectiveness in LCS was addressed by the European Society of Radiology (ESR) and European Respiratory Society (ERS) in an ESR/ERS statement paper in 2020 [6]. It needs to be considered across multiple dimensions, including healthcare systems, patient outcomes and societal impacts.

## Scientific advancement

The survey can drive scientific progress in medical imaging. By identifying factors influencing radiation exposure, translational research can initiate further developments promoting more efficient CT acquisition protocols, balancing image quality with minimal radiation dose via protocol optimization in the technical [7] and personnel realm [8]. But without more detailed knowledge of the currently applied CT acquisition protocols, the addition of densitometry for emphysema or coronary calcium detection to the diagnostic arsenal alongside LCS would be merely wishful thinking. Understanding current practices and limitations can guide the development of new CT technologies and reconstruction algorithms that further reduce radiation exposure.

## Conclusion

By addressing these aspects, a survey on CT acquisition protocols for LCS can contribute to the overall sustainability of healthcare systems and medical imaging practices.

A preliminary survey has been sent out in Europe within the SOLACE consortium. We would like to further advance this effort with a concerted distribution effort across research institutions focused on LCS in Europe and to all active screening centers in the United States to obtain a cross-continental overview of the current status of CT acquisition protocols in LCS.

**Fig. 1:**
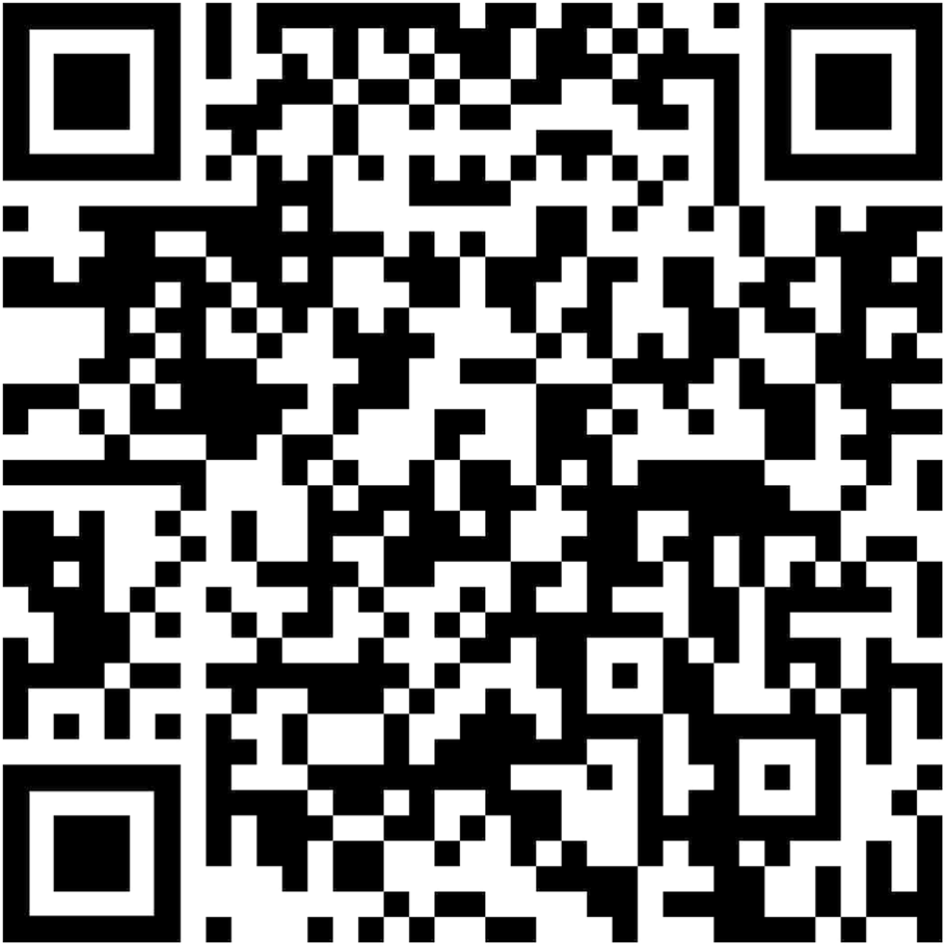
QR code to access a link to the CT acquisition protocol survey website by scanning the code

The survey can be found at the link below or by scanning the QR code in Fig. 1: https://redcap.link/CT_Protocol_LCS.

The survey will remain open for 6 months to allow the invitation to be shared widely, enabling contributions from all relevant institutions. To adhere to the principle of sustainability, the survey will reopen after approximately 3 years to gather an update on the current status of CT acquisition protocols in use for LCS. This longitudinal approach will enable us to track changes over time, assess the impact of standardization efforts (e.g., on artificial intelligence models), and identify emerging trends or challenges in the field. Between surveys, we plan to disseminate findings through publications and conferences, encouraging the adoption of best practices. This iterative process of data collection, analysis, and knowledge sharing aligns with broader sustainability goals in radiology [9] by promoting continuous improvement and resource-efficient practices in LCS.

We would like to bring this forward on behalf of the SOLACE consortium [10] in collaboration with the European Imaging Biomarkers Alliance (EIBALL), the European Society of Thoracic Imaging (ESTI), the Quantitative Imaging Biomarkers Alliance (QIBA) and the American Lung Association (ALA).

## Abbreviations

CT: Computed tomography
ERS: European Respiratory Society
ESR: European Society of Radiology
LCS: Lung cancer screening
NELSON: Dutch-Belgian randomized lung-cancer screening trial
SOLACE: Strengthening the screening of lung cancer in Europe
